# O067. Osmophobia in allodynic migraine: role of frequency of attacks and headache duration

**DOI:** 10.1186/1129-2377-16-S1-A104

**Published:** 2015-09-28

**Authors:** Carlo Lovati, Luca Giani, Elisa Capiluppi, Giulia Preziosa, Domenico D'Amico, Claudio Mariani

**Affiliations:** Headache Center, Neurology Unit, A.O. L. Sacco, Milan, Italy; Headache Center, C. Besta Neurological Institute and Foundation, Milan, Italy

## Background

Migraine is a primary headache with recurrent attacks of head pain with associated symptoms like nausea, phonophobia, and photophobia. Osmophobia, although not included in diagnostic criteria, seems to be a very migraine-specific symptom[1]. Cutaneous allodynia (CA) is also a common symptom in migraine, especially when frequency of attacks is high. CA is considered a clinical manifestation of central sensitization, a mechanism involved in migraine chronification[2]. Recent works put in evidence a relationship between the presence of osmophobia and CA in migraineurs[3]. This study was aimed to investigate possible clinical elements able to influence the relationship between osmophobia and allodynia in migraine.

## Materials and methods

We enrolled 871 patients consecutively evaluated in our Headache Center. Chronic migraine was defined as a mean frequency of headache of at least 15 days per month. Two hundred and sixty-three patients had chronic migraine (63 with aura, ChMA, and 200 without aura, ChMO) and 608 were episodic (165 with aura, MA, and 443 without aura, MO).

## Results

Osmophobia was significantly more frequent among patients with CA with respect to patients without CA (33.9% vs 26.7%, p = 0.016 at Chi square test). The association between these two symptoms was significant only in chronic migraineurs, among which osmophobia was present in 39.4% of allodynic patients and in 24.1% of non-allodynic patients (p = 0.008 at Chi square test). No difference was found in the distribution of osmophobia comparing chronic migraineurs with and without aura (44% of ChMA and 37% of ChMO). Both CA and osmophobia were significantly more frequent among women with respect to men: CA was found in 30% of men and 55% of women (p < 0.001) and osmophobia in 18.2% of men and 32.6% of women (p = 0.001). The relationship between CA and osmophobia was confirmed in both ChMA and ChMO among women. Even if the proportion was similar, significance was not found among men, probably because of the smaller sample size. Osmophobic episodic migraineurs, both with and without aura, had a longer migraine history. This evidence was not found among chronic patients.

## Conclusions

The highlighted relationship between allodynia and osmophobia seems not to be influenced by gender nor by aura. The observation that it is related to a higher frequency of attacks and longer history of migraine may be interpreted as a common consequence of central sensitization, able to induce in parallel a distortion of both cutaneous sensitivity (CA) and olfaction (osmophobia).

Written informed consent to publication was obtained from the patient(s).
